# Biopharmaceutical-Type Chinese Hamster Ovary Cell Cultivation Under Static Magnetic Field Exposure: A Study of Genotoxic Effect

**DOI:** 10.3389/fbioe.2021.751538

**Published:** 2021-11-25

**Authors:** Alina Rekena, Dora Livkisa, Edmunds Kamolins, Juris Vanags, Dagnija Loca

**Affiliations:** ^1^ Rudolfs Cimdins Riga Biomaterials Innovations and Development Centre of RTU, Faculty of Materials Science and Applied Chemistry, Institute of General Chemical Engineering, Riga Technical University, Riga, Latvia; ^2^ Department of Microbiology and Biotechnology, Faculty of Biology, University of Latvia, Riga, Latvia; ^3^ Institute of Physical Energetics, Riga, Latvia; ^4^ Institute of Industrial Electronics and Electrical Engineering, Riga Technical University, Riga, Latvia; ^5^ Bioengineering Laboratory, Latvian State Institute of Wood Chemistry, Riga, Latvia; ^6^ Baltic Biomaterials Centre of Excellence, Headquarters at Riga Technical University, Riga, Latvia

**Keywords:** Chinese hamster ovary cells, static magnetic field, magnetically coupled stirring, bioreactors, biopharmaceuticals, genotoxicity, chromosomal damage, CBMN assay

## Abstract

The lack of a sufficient research base is the reason for the ongoing discussion regarding the genotoxic effect of magnetic field (MF) exposure on mammalian cell cultures. Chinese hamster ovary (CHO) suspension-type cells, which are widely used for biopharmaceutical production, are potentially subjected to an increased MF when cultivated in bioreactors equipped with bottom-placed magnetically coupled stirring mechanisms. The main challenge for conducting research in this field remains the availability of a suitable experimental setup that generates an appropriate MF for the raised research question. In the present study, a simple and cost-effective experimental setup was developed that generated a static MF, similar to what has been modeled in large-scale bioreactors and, at the same time, was suitable for experimental cell cultivation in laboratory conditions. The measured maximum magnetic flux density to which the cells were exposed was 0.66 T. To assess the possible genotoxic effect, cells were continuously subcultivated in laboratory petri dishes for a period of 14 days, corresponding to a typical duration of a biopharmaceutical production process in a conventional fed-batch regime. The genotoxic effect was assessed using the cytokinesis-block micronucleus assay with fluorescent staining. Results showed that a 0.66-T static MF exposure had no significant long-term effect on cell viability and chromosomal damage but demonstrated a short-term effect on cell apoptosis. Significant increase in nuclear bud formation was observed. These findings may encourage other researchers in future studies investigating cellular responses to MF exposure and contribute relevant data for comparison.

## Introduction

Although the magnetic field (MF) influence on various biological systems has been widely reviewed (Dini and Abbro, 2005; Miyakoshi, 2005; Ghodbane et al., 2013; Zhang X. et al., 2017), it is challenging to find sufficient evidence when a case study with defined MF parameters is presented. For example, Potenza et al. (2010) discovered that a 0.3-T static MF for 4 hours induced DNA damage both at the nuclear and mitochondrial levels of human umbilical vein endothelial cells, but recently Wang et al. (2016) reported no changes in internal DNA structure for adipose-derived stem cells after a 0.5-T 7-day static MF exposure. This illustrates the heterogeneity of research often seen in this field and highlighted by authors working in this field (Chionna et al., 2003; Potenza et al., 2010; Tian et al., 2018).

In current research, the MF exposure is discussed in the context of MF-initiated stirring mechanisms (agitators) in bioreactors, as the cells can directly interact with the MF in the case of bottom-placed magnetic coupling mixers. In brief, they are made of the drive and driven magnets interacting at the end of an agitator shaft (Liu, 2013; Stanbury et al., 2017). In this construction, the stirrer axis does not pierce the agitator shaft, instead a small magnetic gap is formed between the shaft and driven magnets with bearings where the cells are exposed to the generated MF (Jagani et al., 2010; Reichert et al., 2012; Ladner et al., 2018). The nature of the MF that this mechanism generates has been reviewed in detail elsewhere (Rekena et al., 2019), but both static and time-varying MFs are generated. It has been reported that in large bioreactors equipped with magnetic coupling–initiated agitation, a magnetic flux density could reach the values from 0.87 to 1.36 T inside the magnetic gap (Orlova et al., 2018). This technology is easier to clean and sterilize and thus could be particularly useful for mammalian cell cultivation as they require the highest level of sterility, compared to other industrial microorganisms (Kretzmer, 2002).

The industrial application of mammalian cell cultures is for the production of biopharmaceuticals (Zhu, 2012). These products are used to therapeutically treat a wide spectrum of diseases, including cancer and autoimmune and genetic disorders (Ghaderi et al., 2012; Kesik-Brodacka, 2018). One of the ways to produce the drugs is to apply a 14-day fermentation process in a stirred-tank bioreactor on a fed-batch regime (Levine et al., 2013; Bunnak et al., 2016; Dorceus et al., 2017; Bausch et al., 2018; Lindskog, 2018). One of the most commonly used cell lines for this purpose is Chinese hamster ovary (CHO) cells (Jayapal et al., 2007; Kim et al., 2012).

CHO cells have been studied under MF exposure of various intensities by several authors. Zhao et al. (2010) investigated the effect of extremely strong (13-T) static MF on CHO cell cycle and viability and reported no effect. Zhang et al. in two subsequent studies investigated the effect of a moderate (1-T) static MF on CHO cell growth for 2 days and reported no effect; however, other cell types in the same study were affected (Zhang et al., 2017). They also reported that CHO cell proliferation changed in response to a strong (9-T) static MF when cells were transformed for protein expression (Zhang et al., 2016). In another study by Tian et al. (2018), CHO cell growth was reduced after 0.2–0.5 T static MF exposure for one night.

The abovementioned results correspond to the opinion by Miyakoshi (2005) who assured that in most cases, but not all, static MF has no influence on cell growth and proliferation regardless of magnetic density . He proposed that the question of whether MF causes genotoxic effect should be a further area of interest.

The potential of cell chromosomal damage is an important factor to be considered during recombinant protein manufacturing as micronuclei (MN), nucleoplasm bridging, and nuclear bud (NBud) formation are known to be induced by chromosomal fragmentation, DNA misrepair, altered gene replication, and other malfunctioning cell mechanisms, indicating chromosomal instability (Fenech, 2006). This can lead to gene (including the recombinant gene of interest) loss and changes in DNA sequences and thus altered transcription and translation of the final products.

As such, a significant increase in cell nuclear abnormalities can mean a decrease in the productivity and efficiency of any technology. The only study, to our best knowledge, on genotoxic effect of CHO cells has been reported by Nakahara et al. (2002), showing no effects on MN formation on CHO cells at strong (10-T) static MF for 4 days.

Several methods can be used to study the cell DNA damage, including stathmokinetic, flow cytometric, and DNA labeling approaches. However, the cytokinesis-block MN (CBMN) assay has been reported to be superior to others (Fenech, 2000) and has been used in previous studies (Nakahara et al., 2002).

One of the challenges for conducting research that involves the application of MF to cells is to choose an appropriate experimental setup. A magnetic yoke from permanent magnets was constructed especially for this experiment. It generated a MF of the desired type and strength and was easy to use for cell cultivation in standard laboratory petri dishes. In the current research, the genotoxic effect on CHO cells during the cultivation under a moderate MF exposure, similar to MF that could be found in large bioreactors equipped with magnetically coupled stirring mechanisms, was investigated.

## Materials and Methods

### Methods for Cell Cultivation

Frozen FreeStyle™ CHO suspension cells (Thermo Fisher Scientific, Carlsbad, United States) were rapidly thawed in 37°C water bath. For inoculation, 30 ml of warm basal medium (BM) in a glass baffled-bottom 250-ml Erlenmeyer flask with a membrane screw cap (Duran®, DWK Life Sciences, Mainz, Germany) was used. BM consisted of FreeStyle™ CHO Expression Medium (Life Technologies, New York, United States) supplemented with 8 mM L-glutamine (200 mM, Gibco®, Life Technologies, Paisley, United Kingdom) and 1% (v/v) Penicillin–Streptomycin antibiotics (Gibco®, Life Technologies, New York, United States). The cell suspension was cultivated in a CO_2_ incubator (New Brunswick™ S41i, Eppendorf, Hamburg, Germany) at 8% CO_2_, 37°C, and 95% humidity with shaking speed of 125 rpm (25 mm orbital shaking diameter) overnight. Afterward, the cell suspension was transferred to two 15-ml tubes and centrifuged at 1,500 rpm for 6 min (Compactstar CS4, VWR, Leuven, Belgium). After discarding of the growth medium, the cell pellet was resuspended in 30 ml of BM and transferred to a new 250-ml flask. Cells were further incubated in the previously described conditions overnight. Afterward the cells were subcultured every 2–3 days (duration of one passage) in the lag phase at 2.0 × 10^5^ viable cells/mL, and the rest was cryopreserved in 10% (v/v) dimethyl sulfoxide (DMSO)/BM in −80°C. DMSO was purchased from Sigma-Aldrich (Sheboygan Falls, Wisconsin, United States).

For the experiments, disposable 92 mm × 16 mm petri dishes (Sarstedt, Numbrecht, Germany) were used to grow suspension cell culture. Cells were inoculated at 0.5 × 10^6^ viable cells/ml in 10 ml of BM. Experiments were performed in duplicate at shaking speed of 80 rpm and subcultured every 2–3 days for a total duration of 14 days. The number of replicates corresponds to the number of independent experiments.

Cells were subcultured regularly with the time interval of one passage (2–3 days). For subculturing purposes, the cell suspension was transferred to a 15-ml tube and centrifuged at 1,500 rpm for 6 min (Compactstar CS4, VWR, Leuven, Belgium). The cell pellet was resuspended in 1–3 ml of fresh BM, and cells were counted using the protocol of trypan blue exclusion method. In brief, 10 μL of the cell suspension was mixed with 10 μL trypan blue stain (Life Technologies, New York, United States), transferred to a hemocytometer (Neubauer Improved Assistent®, Hecht-Assistent, Sondheim/Rhön, Germany), and viable (white) and dead (blue) cells were counted manually using an inverted light microscope DM IL (Leica Microsystems, Wetzlar, Germany). Then cells were inoculated again by diluting at 0.5 × 10^6^ viable cells/mL in 10 ml of warm BM. The rest of the cell pellet was discarded.

### Magnetic Field Exposure

**FIGURE 1:**
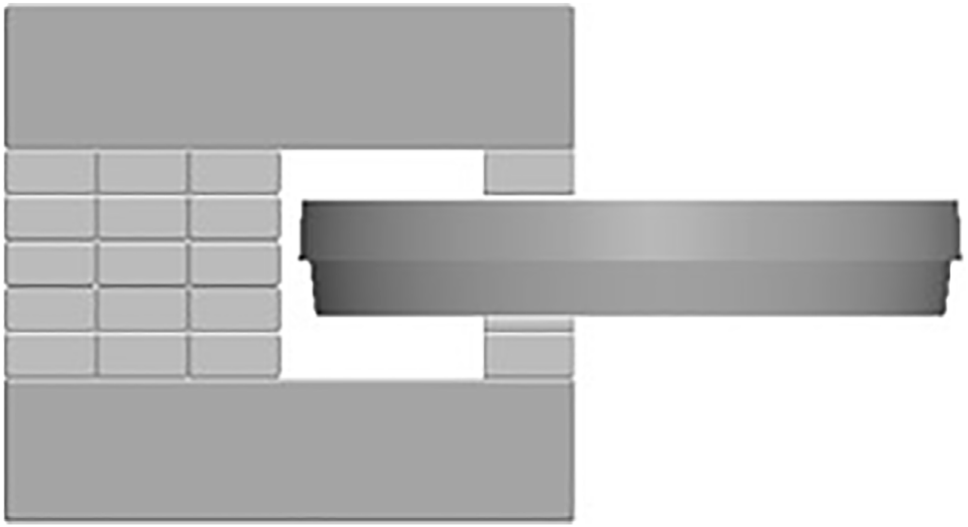
The experimental setup of a static MF exposure: magnetic yoke from neodymium rare-earth magnets NdFeB grade N48H (see Supplementary Figure S1) suitable for a standard 92 mm × 16 mm cell culture petri dish.

In order to expose the cells to a static MF, neodymium rare-earth magnets NdFeB grade N48H 1” × ½” × 1/8” and 1” × ½” × 1/4” (K &J Magnetics, Pipersville, Pennsylvania, United States) and structural steel grade S235JR (Basissteel Group, Smolensk, Russia) were used to make a simple magnetic yoke with an air gap suitable for a standard 92 mm × 16 mm petri dish (Figure 1, Supplementary Figure S1). According to the gauss meter (model 410, Brockhaus, Ludenscheid, Germany) measurements, magnetic flux density inside the air gap of the yoke is from 0.49 ± 0.010 T to 0.74 ± 0.015 T (Supplementary Table S1). The maximum magnetic flux density to which the cells are exposed was measured at the point in the petri dish where cell suspension meets the bottom inner wall of the petri dish, and it is 0.66 ± 0.013 T (Supplementary Figure S2, Supplementary Table S1). The cell culture dish was prepared as previously described and inserted in the air gap of the magnetic yoke and placed immediately in the CO_2_ incubator. A negative control was not subjected to MF exposure. The second petri dish with cells was prepared as described previously and placed in the incubator without a magnetic yoke. The absence of a MF outside the perimeter of the magnetic yoke with an experimental sample, as shown in Figure 1, was confirmed with a gauss meter.

### Cytokinesis-Block Micronucleus Assay

The CBMN assay was performed to assess the genotoxic effect of MF on CHO-S suspension cells. The method was taken from publications by Fenech (Fenech, 2000; Fenech et al., 2003) and adapted during the pilot studies.

The CBMN assay was performed on cell samples at the beginning of the experiment (0 days) and on the 1st, 9th, and 14th day of the experiment. For the analysis, aliquots of 1 × 10^7^ viable cells of MF-exposed samples and negative control samples were transferred from the petri dish, where cells were repeatedly subcultured during the whole period of the experiment (14 days), to a new petri dish (one for each sample) with a final volume of 10 ml of BM. Cytochalasin B from *Drechslera dematioidea* (Sigma-Aldrich, Jerusalem, Israel) with a concentration of 5 μg/ml was added to the cells, and they were incubated for 24 hours without the MF. Then cytochalasin B was removed by centrifugation at 1,500 rpm for 6 min (Compactstar CS4, VWR, Leuven, Belgium) and cells were rinsed with phosphate-buffered saline (PBS) without Ca^2+^ and Mg^2+^ (Dulbecco’s PBS, Gibco®, Life Technologies, Paisley, United Kingdom) and fixed in formaldehyde (Merck, Darmstadt, Germany) 4% (v/v) solution for at least 25 min. The fixator was then removed by centrifugation, and the cells were washed three times with PBS.

The cells were stained with 4′,6-diamidino-2-phenylindole (DAPI) fluorescent dye (AppliChem, Darmstadt, Germany) (0.5 μg/ml) and counted manually by the same operator for all samples using an inverted fluorescent microscope DMI 4000 B (Leica Microsystems, Wetzlar, Germany) with 1000× magnification. Firstly, 500 cells per sample were scored as 1) mononucleated, 2) binucleated (BN), 3) tri-nucleated, 4) tetra-nucleated, and 5) apoptotic and necrotic to calculate the cell viability. The cell viability was calculated using the equation:

$$\text{Viability}=\frac{\text{M}1+\text{M}2+\text{M}3+\text{M}4}{\text{Ap}+\text{Nec}+\text{M}1+\text{M}2+\text{M}3+\text{M}4}$$ (1)

where, M1–M4 are the number of viable cells with 1–4 nuclei, Ap is number of apoptotic cells, and Nec is number of necrotic cells.

Apoptotic and necrotic cells were discriminated based on the morphology of the cell membrane (Fenech et al., 2003). Apoptotic cells were recognized by intact cytoplasmic and nuclear boundaries with nuclear fragmentation into smaller nuclear bodies or the presence of chromatin condensation within the nucleus (early apoptotic cells). Necrotic cells were morphologically discriminated from apoptotic cells by lost cytoplasmic membrane and irregular nuclear membrane often with nuclear material leaking from the nuclear boundary, or vacuolated and pale cytoplasm and the nucleus marginally intact (early necrotic cells). Also, apoptotic cells had greater staining intensity than viable cells, while necrotic cells stained less compared to viable cells.

**FIGURE 2:**
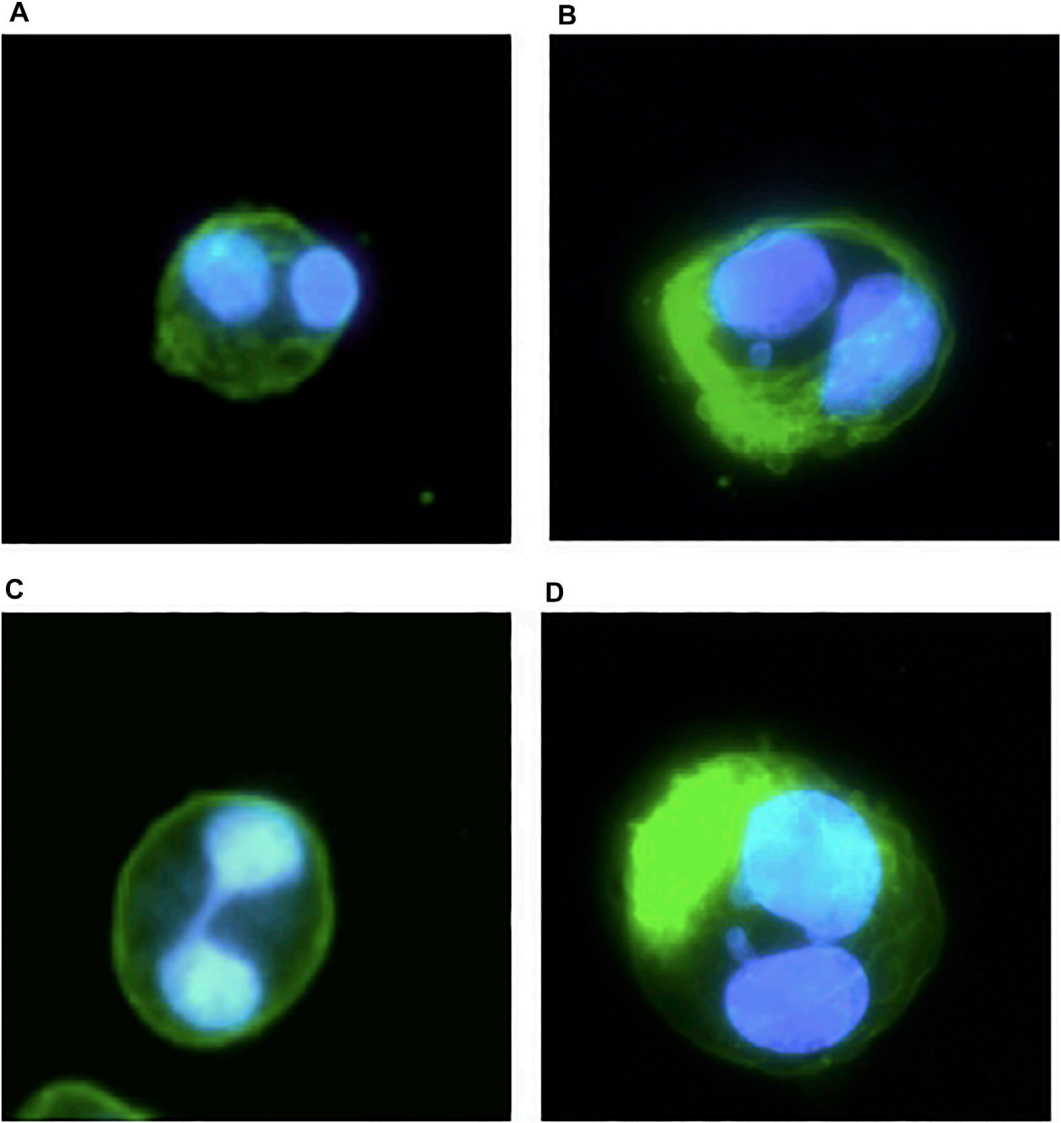
An illustrative example of CHO-S suspension cell microscope images after the CBMN assay taken from the pilot studies and assay protocol validation experiments; blue represents DAPI fluorescent dye staining of the cell DNA for the detection of DNA abnormalities of BN cells: **(A)** normal BN cells; **(B)** BN cells with MN; **(C)** BN cells with NBridge; **(D)** BN cells with NBuds.

Secondly, 1,000 BN cells per sample were scored as 1) normal BN cells, 2) BN cells with MN, 3) BN cells with nucleoplasm bridges (NBridge), and 4) BN with NBuds (Figure 2) to assess the possible genotoxic effect as described by Fenech (Fenech, 2000).

## Results

### Cell Viability After Magnetic Field Exposure

In this study, the effects of DNA damage to CHO-S suspension cell line after exposure to 0.66-T static MF were evaluated by performing the CBMN assay. The cells were treated with cytochalasin B for cytokinesis blocking, then fixed and afterward stained with fluorescent dye to assess the status of nuclei in the cells. During the 14-day cultivation which was designed to correspond to a typical process of biopharmaceutical production in a fed-batch regime, cells were scored to determine cell viability (500 cells per sample) and the accumulation of genotoxic effects (1,000 BN cells per sample).

**FIGURE 3:**
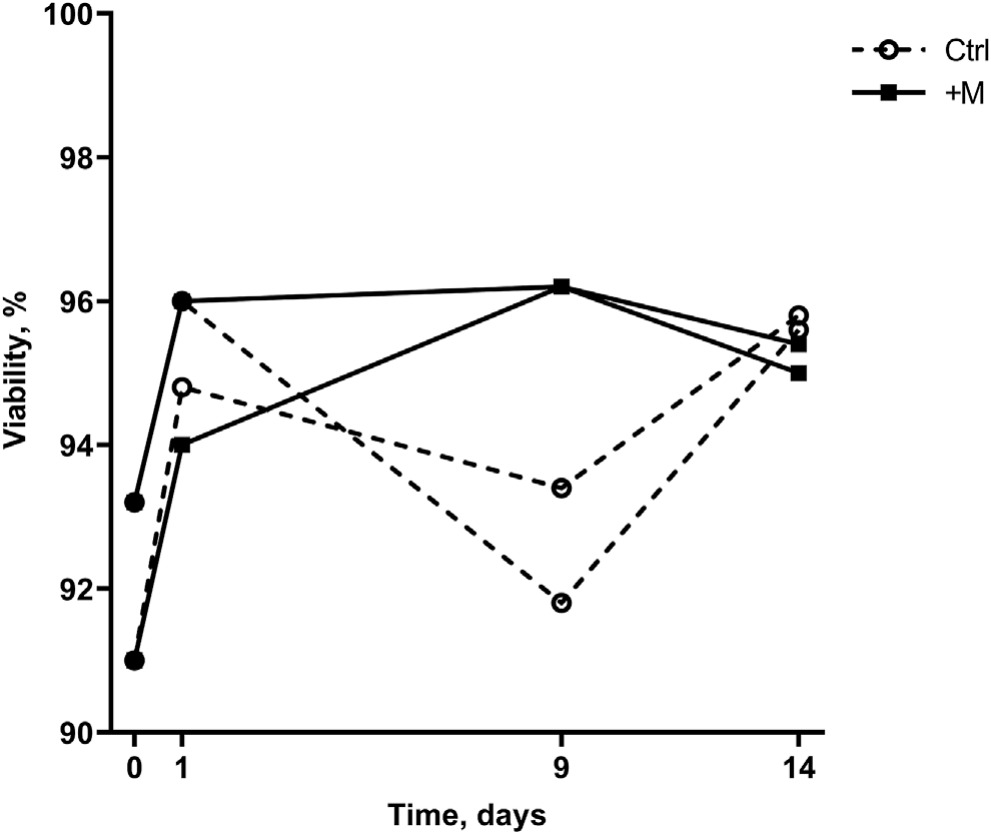
Cell viability of control (Ctrl) and MF-treated (+M) cells cultivated for 14 days.

According to Figure 3, cell viability measurements demonstrated the following two observations. Firstly, the MF exposure affected cell viability only on day 9 when higher viability of MF-treated than control cells was observed. Secondly, the cell viability in MF-exposed samples increased later than in nonexposed cells (day 0 versus day 9 compared with day 0 versus day 1, Figure 3). Overall, cell viability in both groups increased over the whole cultivation period (14 days); on day 0 of the experiment, cell viability was on average 92.1 ± 1.6%, and during the experiment, it increased to on average 95.7 ± 0.1% and 95.2 ± 0.3% on day 14 for negative control (Ctrl) and MF-treated (+M) group, respectively. Interestingly, the maximum viability score for the MF-exposed samples was on day 9 (96.2%), whereas for the control group, cell viability reached a maximum on day 14 (95.7 ± 0.1%). At the same time, the viability of the cells exposed to MF decreased on average by 1% during the period from day 9 to day 14 (from 96.2% to 95.2 ± 0.3%).

### A Closer Look at the Profiles of Dead Cell Numbers

**FIGURE 4:**
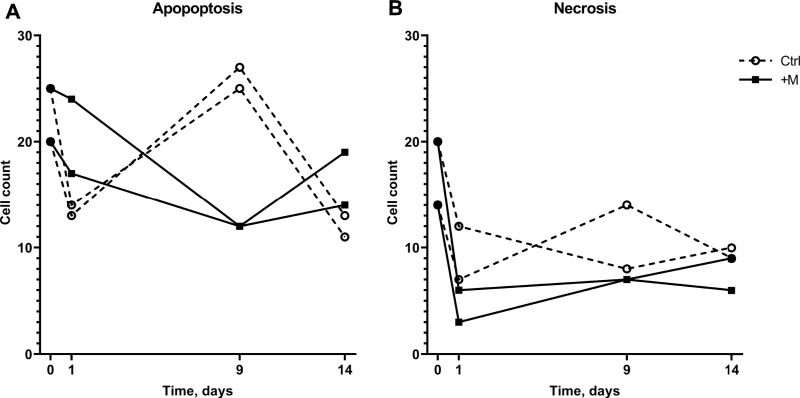
Numbers of apoptotic **(A)** and necrotic **(B)** cells in 500-cell samples of the control and MF-exposed cells cultivated for 14 days.

The effect of MF exposure on cell viability on day 9 (Figure 3) can be explained by more apoptotic cells in the control sample than MF-exposed sample (26 ± 1.4 and 12 ± 0, respectively, Figure 4A, day 9). The change in cell viability can be explained by a decrease in apoptotic cell count in MF-exposed samples that was observed later than in the control samples (day 0 versus day 9 compared with day 0 versus day 1, Figure 4A). Interestingly, in control samples, the cell viability from day 1 to day 9 decreased (Figure 3) due to an increase in apoptotic cell count from day 1 to day 9 (Figure 4A). Overall, more apoptotic than necrotic cells were detected both in the MF-exposed and in the negative control group; in MF-exposed samples more apoptotic than necrotic cells were detected on days 1 and 14, and in the negative control group on day 9 (Figure 4). However, the actual number of cells were quite low (the highest number detected was on average 40 ± 8 dead cells in total at the beginning of the experiment, which corresponds to 8% of the total sample size). The number of necrotic cells in the MF treatment group decreased from day 0 to days 1, 9, and 14.

### The Analysis of Cell Chromosomal Damage

**FIGURE 5:**
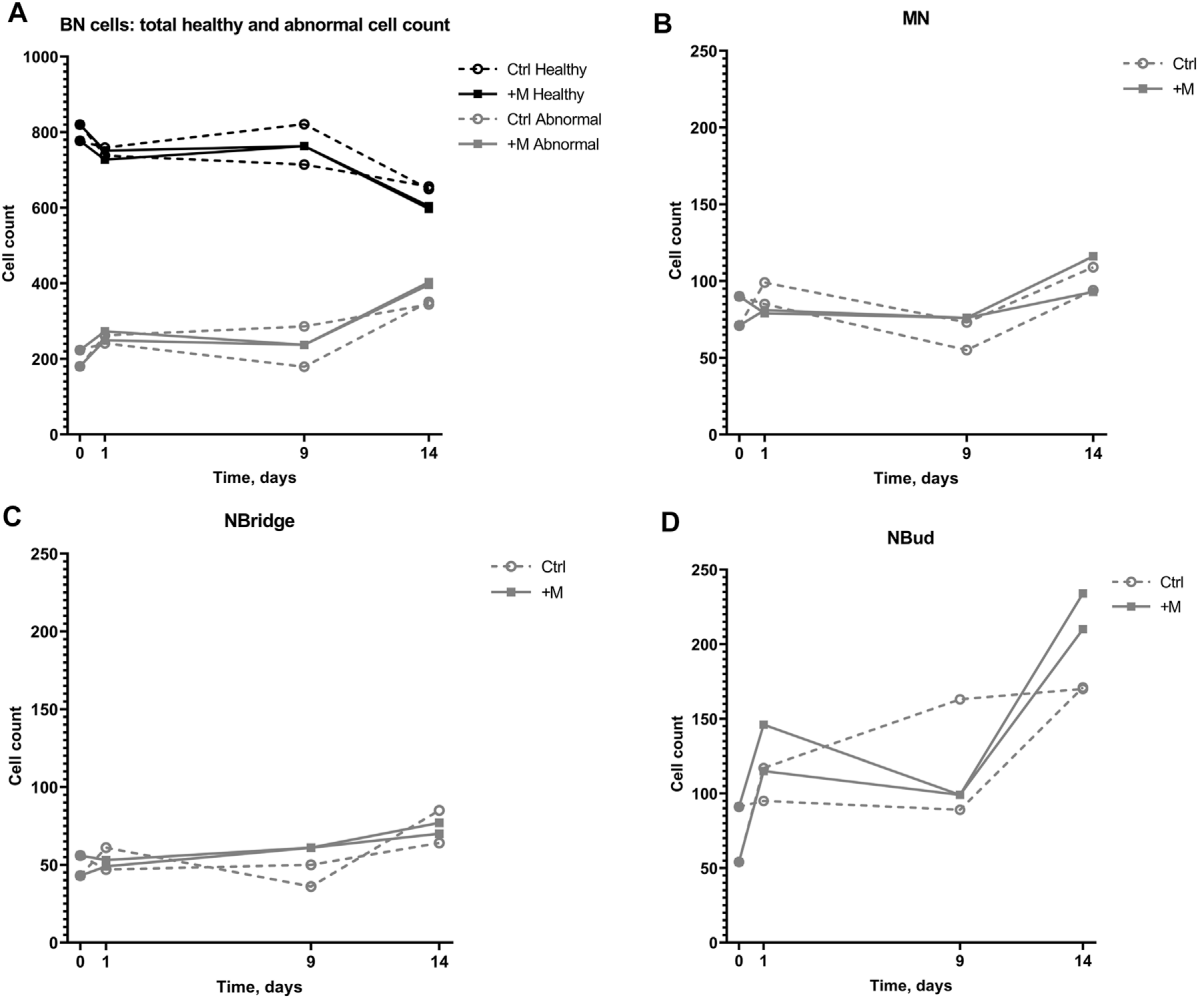
Genotoxic effect on 1000-cell samples of CHO suspension cells cultivated under 0.66-T static MF exposure for 14 days. **(A)** Numbers of binucleated (BN) cells with no chromosomal damage (Healthy) and BN cells with detected chromosomal damage (Abnormal) with (+M) and without (Ctrl) magnetic field exposure; **(B)** numbers of BN cells with MN; **(C)** numbers of BN cells with NBridge; **(D)** numbers of BN cells with NBuds.

The effect of MF exposure was also analyzed in regard to each group of BN cells “health” (Figure 5A), i.e., whether there was no chromosomal damage Healthy or some abnormality was detected (Abnormal). Accordingly, normal BN cells constituted the majority in every 1000-cell sample. Nevertheless, the normal BN cell count decreased over the time of cultivation. In the negative control group, the total number of normal BN cells decreased from 80% down to 60% of the total cell count during the 14-day period. In the MF treatment group (+M), the total number of BN cells without any damage decreased by 25% on an average from 798 ± 30 on day 0 to 601 ± 5 on day 14.

The total number of abnormal cells in both the groups increased over the cultivation period on an average from 202 ± 30 on day 0 to 347 ± 5 and 399 ± 5 on day 14 in the control and MF-treated groups, respectively, including the cells with MN, NBridge, and NBuds (Figure 5A). The number of cells with NBuds constituted most of the abnormal cells and increased on average from 72 ± 26 (7%) to 170 ± 0.4 (17%) in the negative control group, to 222 ± 17 (22%) cells in the treatment group (Figure 5D) over the 14-day cultivation period. An increase in the number of cells with MN was observed from day 9 to day 14 for the control group (on average from 64 ± 13 to 102 ± 11, Figure 5B and Table 1). Similarly, the number of cells with nuclear bridges also increased only from day 9 to day 14 for the control group (43 ± 10 to 75 ± 15, Figure 5C). In the MF treatment group, the highest number of abnormal cells of all types (MN, nuclear bridges, NBuds) were counted on the last day of the experiment (day 14) (104 ± 16, 73 ± 5, and 222 ± 17 cells, respectively, Figures 5B–D). Overall, the duration of the cultivation period had the greatest effect on CHO suspension cell chromosomal damage in both control and MF-exposed samples.

**TABLE 1:** MN formation on 1000-cell samples of CHO suspension cells cultivated under 0.66-T static MF exposure for 14 days and SD.

Samples	MN (of 1,000 cells)	SD
Day 0	80	13
Day 1	92	10
Day 1	80	2
Day 9	64	13
Day 9	76	0
Day 14	102	11
Day 14	104	16

No difference in cell count between the control and MF-exposed samples was observed until day 9 of the experiment (Figure 5A). On day 14 of the experiment, the number of abnormal cells with NBuds was higher under MF exposure than in the negative control group (Figure 5D).

## Discussion

Although controversial experimental data on MF effects on various biological systems could be found in the scientific literature, as noted by Zhang et al. (2017b), the majority of inconsistencies among the research can be explained by the differences in experimental settings of the MF or biological samples used in the studies. As such, it raises many questions about the specific mechanisms involved in the CHO cell responses to the MF. Previously, a similar study was carried out by Nakahara et al. (2002); however, different strength (10 T) and exposure time (4 days) of MF was applied, thus making it difficult to compare the obtained results between both studies.

In general, there seems to be a lack of research on long-term (days to months) MF exposure and impact. It could be considered that there has been no increased interest on this subject because normally biological systems in everyday life are not exposed to MF more than the planets geomagnetic field [25–65 µT (Nakahara et al., 2002)] for a long period of time, but short-time (seconds to hours) and periodic MF exposure is being investigated due to its increasing existence in everyday life (caused by various electrical appliances), use in medicine (e.g., magnetic resonance imaging), and potential for new therapies (e.g., in combination with anticancer drugs).

Currently, the most common explanation for the MF effect is a radical pair mechanism (Lagroye et al., 2011; Zhang X. et al., 2017). Buchachenko (2016) offers a more detailed insight into the chemistry behind it, and points out more specifically MF-dependent and magnetosensitive “radical pair mechanism of the phosphorylation in the three processes of paramount importance, three cornerstones of the life chemistry—enzymatic ATP synthesis, DNA replication, and enzymatic phosphorylation of proteins”. This, in turn, serves as a reason to examine chromosomal stability, as DNA replication includes DNA—and thus also chromosomal—repair. However, most studies on DNA and chromosomal stability have looked at different aspects of DNA damages (single- or double-strand breaks) using different methods (e.g., Comet assay), which makes the comparison of the obtained results complicated, as DNA strand breaks within the nuclei could not be detected by microscopy methods.

Another significant effect of MF is its ability to alter Ca^2+^ concentrations in the cells, where it plays an important role in regulating various cell signaling pathways, including apoptosis (Zhang X. et al., 2017). Depending on the cell type, influx of Ca^2+^ may either inhibit or promote cell apoptosis (Teodori et al., 2002). It has been shown before that apoptosis in CHO cell lines is Ca^2+^ influx dependent (Pigozzi et al., 2004). However, no information on MF effect on these changes in intracellular Ca^2+^ levels could be found.

Firstly, the obtained results demonstrated that MF exposure did not cause long-term differences in cell viability (Figure 3, day 14). However, MF might have a short-term effect, indicating the faster adaptation rate in the control group than in the MF-exposed cells (according to an increase in viability for the control group from day 0 to day 1 and for the MF-exposed group from day 0 to day 9, Figure 3). It has been suggested that MF can potentially inhibit induced cell apoptosis while simultaneously decreasing DNA repair rates (Fanelli et al., 1999; Robison et al., 2002). Cells under MF exposure could be more likely to accumulate structural DNA damage and oxidative stress than self-destruct, while the control group could have a more natural response in the form of apoptosis followed by cell lysis. Data in Figure 4 demonstrate that the number of apoptotic cells in MF-exposed cell samples decreased to a smaller extent than in the control samples from day 0 to day 1. It cannot, however, be explained why a sudden increase in apoptotic cells in the control group on day 9 was observed (Figure 4). Future work could investigate whether changes in the cell lysis rates due to applied MF could give an important insight on possible MF inhibitory effects to Ca^2+^ influx in the CHO cell line, thus demonstrating its importance on cell apoptosis and lysis delays. These investigations could serve as an explanation to the higher apoptotic cell numbers on day 1 (Figure 4).

Increase in cell viability observed in both MF-exposed and control samples could be explained with the initial changes in cultivation environment as the cells were moved from the Erlenmeyer flasks to the petri dishes with a smaller media volume. The cell adaptation period could be presumed as an initiator of the observed effect. Senger and Karim (2003) have reported that shear stress caused by the hydrodynamic forces of different intensities differently affects the cell growth rate, as well as cell metabolism and death rates. Also, links between cell culture process parameters and metabolism, including glycosylation, have been established using recent dynamic metabolic modeling approaches. Erklavec Zajec et al. (2021) reported that lower agitation speed results in higher viable cell density (biomass) production and correspondingly higher nutrients consumption in CHO cell culture over 14 days. Furthermore, the model was also used to describe the effect of varying agitation on glycosylated protein production, and the results suggested positive correlation (i.e., lower agitation speed is related to lower number of proteins per cell).

Secondly, results of the present study demonstrated that BN cells with NBuds increased more, compared to all other specific chromosomal damage types over the 14-day cultivation period (Figure 5D). NBud formation is thought to be related to DNA over-replication and consists of amplified DNA localized in the periphery of the nucleus for elimination during the next mitosis (Fenech, 2006). From these results, keeping in mind that the NBud formation increased as well in cells without MF exposure, it can only be speculated that the MF decreased DNA repair as suggested in earlier publications (Fanelli et al., 1999; Robison et al., 2002).

Concerning cell cultivation in bioreactors equipped with magnetically coupled stirring mechanisms, this newly gained data indicates the potential complications in recombinant protein production using permanently modified cell culture strains, as they already tend to be unstable by themselves. Nevertheless, more data should be produced on MF effects not only on the cell cultures used in stable recombinant protein expression systems but also on plasmids and other transfection vectors used in the transient expression systems. These elements of recombinant protein expression systems are also used for biopharmaceutical production and thus are subject to MF exposure in the context of bioreactors equipped with magnetically coupled stirring mechanisms.

## Conclusion

This study investigated the genotoxic effect of a static MF similar to those found in bioreactors equipped with bottom-placed magnetically coupled stirring mechanisms (0.66 T) that are used for the industrial production of biopharmaceuticals. Long-term exposure to a 0.66-T static MF for up to 14 days, which is the typical duration of a biopharmaceutical production process in a conventional fed-batch regime, did not affect CHO cell viability and did not cause significant cell chromosomal damage compared to nonexposed cells (negative control). However, an increase in the number of cells with NBuds was observed in both MF-exposed and control samples (from 7% at the start of experiment to 17 and 22% after 14 days, respectively) which will require further investigation to eliminate the possibility of any effect of MF exposure. In addition, cell viability analysis demonstrated a short-term effect of MF exposure. A later decrease in dead cell numbers (i.e., later increase in viability) was observed in MF-exposed samples, possibly related to the inhibition of induced cell apoptosis and altered cell signaling pathways via Ca^2+^ influx pathways in the presence of MF.

It is possible that the short-term effects on cell viability as a result of MF exposure might have consequences for target protein production. However, in bioreactors equipped with magnetically coupled stirrers, the cells are not constantly located in the gap between the shaft and driven magnets of the stirring mechanism but move around in the bioreactor. Therefore, the effect on cell metabolism in large, industrial-scale bioreactors should be smaller than demonstrated by the results in this study.

## Data Availability

The raw data supporting the conclusions of this article will be made available by the authors, without undue reservation.
