# Supplementary material for: Biopharmaceutical-Type Chinese Hamster Ovary Cell Cultivation Under Static Magnetic Field Exposure: A Study of Genotoxic Effect
Source: Front Bioeng Biotechnol. 2021 Nov 25;9:751538. doi: 10.3389/fbioe.2021.751538 (PMC8656418; doi:10.3389/fbioe.2021.751538)
Supplement: Supplementary file 1 [file DataSheet1.docx]

Supplementary Material

# Supplementary Figures


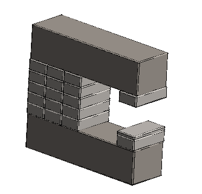


1

1

2

3


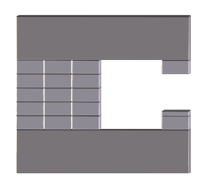


B

A

**Supplementary Figure 1.** Laboratory magnetic yoke: (A) side view, where 1 – steel grade S235JR; 2 – magnet NdFeB N48H (1’’ x ½” x 1/8”); 3 – magnets NdFeB N48H (1’’ x ½” x 1/4”); (B) projection view.

B

A


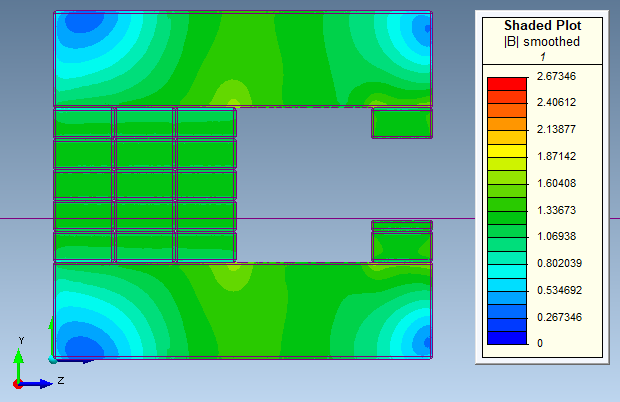

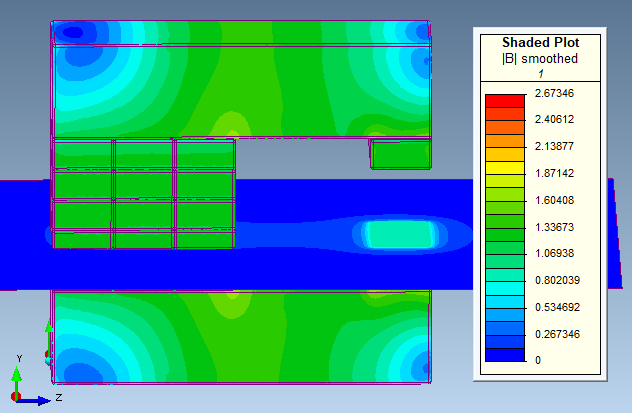


**Supplementary Figure 2.** Mathematical modelling (Mentor MagNet 2D/3D) of the distribution of magnetic flux density of the magnetic yoke with a measuring plane located where the cell suspension meets the bottom inner wall of a Petri dish: (A) side view; (B) projection with the measuring plane.

# Supplementary Tables

| **Parameter description** | **Mathematical modelling, T** | **Experimental with gaussmeter, T** | **Difference, %** |
| --- | --- | --- | --- |
| Maximum magnetic flux density: on the bottom of the air gap on the surface of magnets | 0.83 | 0.74±0.015 | -10.4 % |
| Maximum magnetic flux density: on the bottom of the air gap 0.35 mm (thickness of a bottom inner wall of a Petri dish) above the surface of magnets - at the contact surface of the cell suspension and the bottom inner wall of a Petri dish, see Supplemental Fig. 2. | 0.72 | 0.66±0.013 | -8.3 % |
| Minimum magnetic flux density: in the middle of the air gap | 0.51 | 0.49±0.010 | -4.7 % |

**Supplementary Table 1.** Magnetic field measurements.
